# Dental Care Use Among Pregnant Women in the United States Reported in 1999 and 2002

**Published:** 2004-12-15

**Authors:** Paul I Eke, Peggy Timothé, Scott M Presson, Dolores M Malvitz

**Affiliations:** Surveillance, Investigations, and Research Team, Division of Oral Health (DOH), Centers for Disease Control and Prevention (CDC); formerly research fellow of the Association of Schools of Public Health, CDC, Chamblee, Ga; Program Services Team, DOH, CDC, Chamblee, Ga; Surveillance, Investigations, and Research Team, DOH, CDC, Chamblee, Ga

## Abstract

**Introduction:**

The purpose of this study was to determine national and state-specific estimates of dental care use among adult pregnant women in the United States using data from two 12-month periods. The study also determined person-level characteristics that may predict a lack of dental care use within this subgroup.

**Methods:**

Responses were analyzed from 4619 pregnant women aged 18 to 44 years who participated in the 1999 and 2002 state-based Behavioral Risk Factor Surveillance System. Dental care use was defined as having a dental visit or a dental cleaning in the 12 months preceding the interview. State-specific estimates were adjusted to the 2000 U.S. population distribution. Multivariable regression analysis was used to evaluate person-level characteristics that may predict not obtaining dental care during this period.

**Results:**

Overall, 70% of pregnant women in 1999 and 2002 had received dental care in the previous 12 months. Age-adjusted estimates ranged from 36% (Nevada) to 89% (Vermont) to 91% (Puerto Rico). In 19 states, 75% or more of pregnant women had obtained dental care in the previous 12 months (age-adjusted figure). Most pregnant women with dental care were non-Hispanic white and married, and they had a greater than high school education. Income and smoking status were significant predictors for not using dental care.

**Conclusion:**

In several states, more than 70% of pregnant women reported a dental visit or dental cleaning during the previous 12 months. Relative to the general population, pregnant women are as likely to receive dental care, but certain subgroups need to do much better. However, these estimates may be biased toward a population with a higher socioeconomic status and may not represent dental care use among pregnant women in the general U.S. population.

## Introduction

An estimated 6 million women in the United States become pregnant each year (1). Although preventive dental care (e.g., dental cleaning) will improve the overall health of pregnant women and may reduce their risk of adverse pregnancy outcomes, women who are pregnant are known to use dental services less frequently and at lower levels than the general population (2-4). An interrelated set of financial, personal, and social barriers have been identified as possible reasons why subgroups in most need of dental care may be less likely to receive dental care services (5).

Evidence is increasing that poor oral health may be associated with adverse pregnancy outcomes. Several observational studies have reported associations between periodontal infections and increased risk for poor birth outcomes, such as preterm labor or premature rupture of membranes (6-8). These findings are further supported by experimental animal studies that found maternal exposure to periodontal pathogens resulted in abnormal fetal outcomes (9,10). Preliminary findings from intervention studies also suggest that treatment of advanced periodontal infections may reduce the risk of adverse birth outcomes (11,12).

Currently, information is limited at the national and state levels on patterns of dental care use, particularly dental cleaning, among pregnant women. The current literature is limited to estimates from five states (Louisiana, Illinois, New Mexico, Arkansas, Washington) participating in the Pregnancy Risk Assessment Monitoring System (PRAMS); the proportion of new mothers who received dental care during their most recent pregnancy ranged from 23% to 58% in these five states (13,14).

The purpose of the present study was to determine national and state-specific estimates of dental care use (i.e., having a dental visit or a dental cleaning) during two 12-month periods among pregnant women aged 18 to 44 years in the United States. These estimates were generated after combining data obtained in 1999 and 2002 by the Behavioral Risk Factor Surveillance System (BRFSS). In addition, this study examined person-level characteristics that predicted not obtaining dental care during this period.

## Methods

The BRFSS is a random, state-based telephone survey of major health risk behaviors, clinical preventive health practices, and health care access that relies on a representative sample of noninstitutionalized adults (aged >18) in the 50 states, District of Columbia, Guam, Puerto Rico, and the U.S. Virgin Islands. Details of the survey are available elsewhere (15).

All female participants (aged ≤44 years) in the BRFSS survey are asked about their pregnancy status with the question, "To your knowledge, are you now pregnant?" In 1999 and 2002, three oral health questions were included in the core module, asked of all participants: 1) "How long has it been since you last visited a dentist or a dental clinic for any reason?" 2) "How many of your permanent teeth have been removed because of tooth decay or gum disease?" and 3) "How long has it been since you had your teeth cleaned by a dentist or dental hygienist?" In the present study, dental care use was defined as having either a dental visit or dental cleaning within the preceding 12 months. BRFSS data for 1999 and 2002 were pooled to increase the samples of pregnant women at the state levels. Analysis was restricted to the dentate, and missing data or persons not responding to the questions were removed from the denominator (<1%). The average nonresponse rate combined across the various characteristics examined in this analysis was 0.80%.

Analysis by SUDAAN (Research Triangle Institute, Triangle Park, NC) (16) was used to account for the complex sampling design of the survey and sampling weights. In separate analyses, estimates were age-adjusted based on the U.S. census population distribution of persons aged ≥18 years in 2000 (17) to provide a sounder basis for comparing estimates among states (18). Estimates of dental care use by pregnant women were stratified by age, level of education, diabetes status, health insurance status, income, marital status, smoking status, and race/ethnicity (Hispanic is a category of ethnicity that may include women of all races). Logistic regression modeling was used to examine characteristics that were significant predictors of pregnant women *not* receiving dental services within the preceding 12 months, adjusting for other potential explanatory variables. Covariates in the model were selected a priori based on previous evidence that the variable was associated with dental care use and that measures of the variable were available in BRFSS.

## Results

National and state estimates for dental care use in the past 12 months among pregnant and nonpregnant dentate women are shown in Table 1. The national estimate for pregnant women having a dental visit or cleaning in the previous 12 months, age-adjusted to the 2000 population, was 70.03% (SE = 1.46%), with state percentages ranging from 48.32% (Nevada) to 87.02% (Vermont). Estimates for nonpregnant women ranged from 62.77% (Texas) to 84.14% (Connecticut). When age-adjusted to the 2000 population, estimates ranged from 36.16% (Nevada) to 91.34% (Puerto Rico). In 19 states, the age-adjusted estimates were 75% or greater.

The distribution of age, race/ethnicity, marital status, education, household income, health insurance status, and smoking status was similar when all pregnant women were compared with those receiving dental care in the past 12 months (Table 2). Most pregnant women reporting a dental visit or cleaning in the preceding 12 months were non-Hispanic white, married, between the ages of 20 and 34 years, and educated beyond high school. In addition, most had health insurance (90.92%, SE = 0.91).

Compared with pregnant women who received dental services, those not receiving dental care were more likely to be aged 20 to 34 years, be active smokers (smoking every day or some days), have less than a high school education, and have diabetes (excluding gestational diabetes). These respondents were also less likely to be married and to have health insurance. Pregnant women who reported not having had dental care in the preceding 12 months were twice as likely to lack health insurance and to use public health clinics or hospital outpatient services. In multivariable logistic modeling, only household income and smoking were significant predictors for not reporting dental services in the previous 12 months (Table 3).

## Discussion

This study reports the first national and state estimates for dental care use during two 12-month periods among pregnant women in the United States. Estimates were obtained from a representative sample of pooled data from the BRFSS in 1999 and 2002. For most states, the BRFSS is the only source of information on dental care use and risk factors for chronic diseases. Analyzing this combined dataset, we find about 70% of pregnant women in the U.S. had either a dental visit or a dental cleaning within the preceding 12 months. This estimate was similar to estimates for the general U.S. population (BRFSS 2002 estimate 70.8%).

In the general population, behaviors related to use of dental care are known to be related to demographic characteristics such as level of education and ethnicity (2,19). Among personal characteristics of pregnant women examined in this study, only income and smoking status were significant predictors for *not* obtaining dental care in the previous 12 months, suggesting that low-income pregnant women may be at higher risk for not receiving dental care. This finding is consistent with reports from PRAMS that pregnant women receiving no dental care were more likely to use tobacco (14). Because low-income women are more likely to smoke, smoking in this subpopulation may be a proxy for low income. Pregnant women who did not receive dental care were skewed toward a younger age, probably because younger women are more likely to have lower incomes. The BRFSS does not collect information on dental insurance, parity, or perceived fears of harm to the fetus, which are important determinants in whether pregnant women obtain dental care. Previous reports also suggest that low-income pregnant women are less likely than their higher-income counterparts to visit the dentist (20). We found that 95% of pregnant women who reported a dental visit in the previous 12 months also had a dental cleaning during that period.

State-specific estimates of dental care use in the previous 12 months among pregnant women varied greatly and generally followed the dental use pattern of the overall population of women aged 18 to 44 in each state. We found relatively higher percentages in states or territories with aggressive preventive dental care programs for pregnant women, such as Puerto Rico. Lower estimates for pregnant women were seen in states such as Virginia, Nevada, and Arkansas and were consistent with lower estimates of dental care use in the general population of these states. It is unclear what factors most influence variation by state. However, the number of community centers with a dentist or dental health program is an important explanatory factor for dental care use among low-socioeconomic status (SES) populations.

Importantly, these estimates do not represent the percentage of women reporting dental care use while pregnant. Depending on the term of pregnancy when surveyed by the BRFSS, there would be a period in the 12 months preceding the interview when women were not pregnant. Health care providers and dentists treat women differently according to pregnancy status, and pregnant women seek dental care differently. A relatively higher or lower percentage of dental visits or cleaning when not pregnant would skew these estimates up or down, respectively.

Notably, state-specific estimates from this study were higher than those published previously from the five states that participated in PRAMS, which ranged from 23% to 58% (11,14). Several factors may account for these differences. First, in PRAMS, questions on use of dental care were restricted to the period when pregnant. Second, while the BRFSS included only adults (i.e., those aged ≥18 years), PRAMS includes all pregnant women (i.e., including those <18 years) and over-samples two or three characteristics, typically low SES. Finally, the BRFSS is a telephone survey and probably includes a higher SES population than PRAMS.

Some limitations should be noted in the use of the BRFSS to obtain estimates for dental care use among pregnant women. First, the survey is based on self reports, which can be influenced by recall bias. Self-reported dental care, however, has been found to be a valid measure for dental care use given adequate sample size and study design (21). Second, the BRFSS is a telephone survey that generally excludes women without residential phones; therefore, the survey might exclude persons of lower SES or households with only cellular phones. Finally, because a relatively small percentage of women are pregnant at any time, samples for pregnant women in most states often were small, sometimes less than 50. We pooled data for 1999 and 2002 to increase the samples and improve estimate reliability, but even then, samples for Maine, Mississippi, and the District of Columbia were small (less than 50), and so estimates from these states may be considered less reliable.

Because preventive dental care may reduce risk for adverse pregnancy outcomes, we must assess how current patterns of dental care use among pregnant women compare with those of the general population. Estimates from this study suggest that dental care use in the previous 12 months among pregnant women is about the same in the general population; in both populations, indicators of SES appear to be important predictors of not using services for those persons (approximately 30%) who have not recently had any dental care. However, we note that lack of health insurance, use of public health clinics, and the use of hospital outpatient services were twice as likely among pregnant women not reporting dental care. One approach to reduce lack of dental care among pregnant women may include providing health insurance. Additionally, health care providers in these health care settings are more likely to come in contact with pregnant women who do not receive dental care. This may present an opportunity to provide important oral health education to these pregnant women.

Barriers to obtaining dental cleaning need to be explored further and be better understood. One approach to addressing dental care use could involve prenatal and professional education on the importance of dental care and the adverse effects of smoking during pregnancy. Overall, these estimates provide baseline information on dental visits and cleaning among pregnant women in the United States and may be useful in formulating oral health policies and programs to improve the health and well-being of pregnant women.

## Figures and Tables

**Table 1:** Distribution of Dental Care Use Among U.S. Pregnant Women Aged 18–44, Behavioral Risk Factor Surveillance System, 1999 and 2002

**State**	**Pregnant, Used Dental Care** **% (95% Confidence Interval)**	**Pregnant, Used Dental Care (Age- Adjusted)** **% (95% Confidence Interval)**
Alabama	73.76 (59.5-88.1)	72.63 (51.4-93.8)
Alaska	67.64 (53.3-81.9)	65.40 (48.9-81.9)
Arizona	53.60 (34.0-73.2)	48.41 (30.0-66.8)
Arkansas	60.47 (47.1-73.8)	57.07 (27.6-88.0)
California	75.64 (67.0-84.3)	73.15 (63.4-83.0)
Colorado	67.41 (55.7-79.2)	64.66 (51.8-77.6)
Connecticut	76.86 (66.5-87.2)	75.06 (62.0-88.2)
Delaware	83.38 (70.2-96.5)	85.48 (75.7-95.3)
District of Columbia	85.91 (74.9-96.9)^a^	83.71 (70.8-96.6)^a^
Florida	72.11 (63.1-81.1)	46.49 (37.3-55.7)
Georgia	79.33 (68.7-89.9)	82.54 (72.5-92.5)
Hawaii	72.83 (59.5-86.2)	72.77 (57.9-87.7)
Idaho	70.52 (61.1-79.9)	69.91 (53.8-86.0)
Illinois^b^	76.65 (67.0-86.3)	75.82 (65.0-86.6)
Indiana	64.94 (50.2-79.6)	56.86 (39.1-74.7)
Iowa	73.85 (62.3-85.4)	77.44 (63.5-91.3)
Kansas	70.84 (61.4-80.2)	73.44 (62.2-84.6)
Kentucky	70.10 (53.2-87.0)	58.46 (40.9-76.1)
Louisiana	82.93 (75.7-90.2)	83.56 (75.4-91.8)
Maine	79.43 (63.4-95.5)^a^	86.45 (76.3-96.7)^a^
Maryland	75.49 (65.1-85.9)	73.82 (59.5-88.1)
Massachusetts	75.06 (66.0-84.1)	76.62 (67.6-85.6)
Michigan	79.73 (70.0-88.7)	74.93 (63.5-86.3)
Minnesota	80.67 (73.6-87.7)	81.22 (73.6-88.8)
Mississippi	67.07 (51.6-82.6)^a^	60.35 (36.7-84.1)^a^
Missouri	64.02 (50.7-77.3)	64.12 (44.5-83.7)
Montana	74.67 (60.0-89.4)	75.95 (60.3-91.7)
Nebraska	74.38 (64.6-84.2)	80.10 (72.3-87.9)
Nevada	48.32 (30.3-66.4)	36.16 (19.0-53.4)
New Hampshire	71.06 (55.6-86.5)	74.30 (60.8-87.8)
New Jersey	80.18 (66.9-93.5)	81.45 (68.4-94.6)
New Mexico	63.48 (51.1-75.8)	66.35 (49.7-83.1)
New York	70.97 (59.4-82.5)	71.59 (58.7-84.5)
North Carolina	70.54 (58.0-83.1)	77.82 (67.6-88.0)
North Dakota	73.24 (60.1-86.4)	56.43 (36.0-76.8)
Ohio	75.91 (63.6-88.3)	70.35 (53.2-87.6)
Oklahoma	68.70 (58.9-78.5)	64.88 (52.2-77.6)
Oregon	68.78 (55.6-81.9)	64.44 (44.8-84.0)
Pennsylvania	81.71 (73.5-89.9)	83.77 (73.4-94.2)
Rhode Island	80.25 (67.5-93.0)	80.95 (69.4-92.6)
South Carolina	75.65 (63.7-87.6)	83.70 (75.9-91.5)
South Dakota	73.78 (63.8-83.8)	74.91 (63.7-86.1)
Tennessee	71.76 (59.0-84.5)	71.94 (57.4-86.4)
Texas	66.02 (57.0-75.0)	65.27 (52.4-78.2)
Utah	75.85 (66.1-85.7)	66.18 (45.6-86.8)
Vermont	87.02 (78.6-95.4)	89.06 (80.5-97.7)
Virginia	56.02 (41.3-70.7)	51.51 (37.2-65.8)
Washington	71.73 (62.7-80.7)	69.53 (59.3-79.7)
West Virginia	73.47 (59.8-87.2)	77.04 (61.3-92.7)
Wisconsin	84.42 (74.6-94.2)	75.41 (59.5-91.3)
Wyoming	60.36 (43.3-77.4)	58.86 (39.9-77.9)
Guam^c^	59.83 (24.2-95.5)^a^	79.58 (61.0-98.2)^a^
Puerto Rico	86.31 (77.5-95.1)	91.34 (85.8-96.8)
Virgin Islands^c^	72.52 (53.9-91.1) a	76.53 (59.1-93.9)^a^
United States	71.16 (69.0-73.3)	70.03 (67.1-72.9)

a Sample size <50.

b Estimate based on half of sampled population because the state used the dual questionnaire method.

c Estimates from 2002 survey only.

**Table 2:** Distribution of Person-level Characteristics for U.S. Pregnant Women Aged 18-44, Behavioral Risk Factor Surveillance System, 1999 and 2002

**Characteristic**	**All** **% (SE)**	**Used Dental Care** **% (SE)**	**Did Not Use Dental Care** **% (SE)**

**Age (years)^a^**	n=4619	n=3393	n=1226

18-19	8.49 (0.87)	8.74 (1.06)	7.88 (1.53)
20-24	26.29 (1.19)	23.99 (1.36)	31.96 (2.37)
25-29	27.37 (1.05)	28.37 (1.27)	24.90 (1.85)
30-34	23.40 (0.99)	25.16 (1.22)	19.07 (1.64)
35-39	11.24 (0.74)	11.24 (0.84)	11.27 (1.53)
40-44	3.20 (0.45)	2.50 (0.35)	4.92 (1.32)

**Race/ethnicity**	n=2684	n=1979	n=705

Non-Hispanic white	60.71 (1.83)	62.92 (2.17)	55.33 (3.23)
Non-Hispanic black	11.19 (1.23)	10.05 (1.29)	13.96 (2.76)
Other non-Hispanic	6.09 (0.87)	5.15 (0.97)	8.39 (1.83)
Multi non-Hispanic	1.49 (0.37)	1.60 (0.48)	1.23 (0.51)
Hispanic (includes all races)	20.51 (1.81)	20.27 (2.18)	21.09 (3.08)

**Marital status^a^**	n=4612	n=3389	n=1223

Married	71.30 (1.24)	73.90 (1.39)	64.88 (2.34)
Divorced	2.97 (0.34)	2.17 (0.29)	4.94 (0.91)
Widowed	0.15 (0.06)	0.14 (0.08)	0.18 (0.10)
Separated	1.42 (0.23)	1.37 (0.24)	1.53 (0.55)
Never married	17.18 (0.99)	16.16 (1.14)	19.71 (1.96)
Unmarried couple	6.97 (0.76)	6.25 (0.79)	8.77 (1.54)

**Education level^a^**	n=4619	n=3393	n=1226

Less than high school	14.47 (1.14)	12.64 (1.27)	18.99 (2.10)
High school	28.31 (1.19)	27.60 (1.39)	30.05 (2.30)
Greater than high school	57.22 (1.34)	59.76 (1.58)	50.96 (2.42)

**Annual household income ($)^a^**	n=4055	n=2989	n=1066

<10,000	5.25 (0.60)	4.95 (0.72)	6.01 (1.06)
10,000-14,999	6.46 (0.81)	5.74 (1.02)	8.29 (1.30)
15,000-19,999	10.43 (0.94)	9.36 (1.14)	13.13 (1.76)
20,000-24,999	9.75 (0.78)	8.57 (0.85)	12.73 (1.79)
25,000-34,999	15.98 (0.97)	14.41 (1.05)	19.95 (2.11)
35,000-49,999	17.76 (0.98)	18.34 (1.10)	16.27 (1.98)
50,000-74,999	16.84 (0.90)	18.84 (1.11)	11.77 (1.55)
75,000 or more	17.54 (0.93)	19.79 (1.12)	11.84 (1.58)

**Diabetes (not gestational)**	n=4417	n=3249	n=1168

Yes	1.08 (0.25)	0.75 (0.18)	1.91 (0.77)
No	98.92 (0.25)	99.25 (0.18)	98.09 (0.77)

**Health insurance status^a^**	n=4613	n=3390	n=1223

Yes	88.37 (0.84)	90.92 (0.91)	82.07 (1.84)
No	11.63 (0.84)	9.08 (0.91)	17.93 (1.84)

**Smoking status^a^**	n=4612	n=3388	n=1224

Yes (every day)	8.74 (0.72)	7.82 (0.78)	11.02 (1.54)
Yes (some days)	3.02 (0.42)	2.61 (0.49)	4.05 (0.83)
Former	21.66 (1.03)	20.93 (1.13)	23.46 (2.06)
Never	66.57 (1.18)	68.64 (1.32)	61.47 (2.37)

**Health care access^a^**	n=4617	n=3390	n=1223

Doctor’s office	74.5(1.6)	79.2(1.8)	61.9(3.5)
Public health clinic	8.0(1.1)	6.2(1.1)	12.8(2.6)
Hospital outpatient	3.4(0.9)	2.3(0.5)	6.3(2.9)
Hospital emergency room	4.3(0.8)	3.7(0.9)	5.9(1.5)
Urgent care center	2.7(0.7)	2.6(0.8)	2.9(0.9)
Some other kind of place	1.3(0.5)	1.8(0.4)	2.4(0.8)
No usual place	3.9(3.0)	3.0(0.6)	6.3(1.3)

a Significant at P ≤ .05, based on chi-square test for independence of association between characteristic and dental care use among pregnant women.

**Table 3:** Possible Predictors for U.S. Pregnant Women Not Having a Dental Visit or Cleaning (N=2226)^a^

**Characteristics**	**Odds Ratio (95% CI)**	***P***

**Age (years)**		

⩽19	0.32 (0.10-0.99)	.37
20-24	0.86 (0.39-1.91)	
25-29	0.84 (0.38-1.83)	
30-34	0.75 (0.34-1.69)	
35-39	0.78 (0.31-1.97)	
40-44	1.00 (ref)	

**Race/ethnicity**		

Non-Hispanic white	1.06 (0.57-1.96)	.09
Non-Hispanic black	1.71 (0.81-3.62)	
Other Non-Hispanic races	1.77 (0.70-4.52)	
Non-Hispanic multiracial	0.26 (0.06-1.26)	
Hispanic	1.00 (ref)	

**Marital status**		

Divorced	1.74 (0.83-3.67)	.29
Widowed	0.08 (0.01-1.03)	
Separated	0.91 (0.30-2.73)	
Never married	1.05 (0.63-1.75)	
Unmarried couple	1.04 (0.53-2.02)	
Married	1.00 (ref)	

**Education**		

<High school	0.63 (0.35-1.34)	.27
High school	1.13 (0.76-1.69)	
⩾College	1.00 (ref)	

**Annual income ($)**		

<10,000	0.65 (0.26-1.61)	.047
10,000-14,999	1.34 (0.57-3.19)	
15,000-19,999	1.75 (0.70-4.37)	
20,000-24,999	1.29 (0.62-2.68)	
25,000-34,999	1.43 (0.78-2.60)	
35,000-49,999	1.43 (0.80-2.56)	
50,000-74,999	0.76 (0.43-1.36)	
>75,000	1.00 (ref)	

**Diabetic status**		

Yes	2.49 (0.72-8.54)	.15
No			1.00 (ref)

**Health insurance**		

Yes	0.69 (0.42-1.14)	.15
No			1.00 (ref)

**Where you get health care**		

Doctor’s office	0.43 (0.19-1.00)	.08
Public health clinic or community health center	0.92(0.35-2.43)	
Hospital outpatient	1.37 (0.38-4.91)	
Hospital emergency room	0.88 (0.29-2.67)	
Urgent care center	0.65 (0.22-1.87)	
Other kind of place	0.50 (0.16-1.63)	
Don’t know	0.96 (0.19-4.82)	
No usual place	1.00 (ref)	

**Smoking**		

Current smoker (every day)	1.53 (0.92-1.14)	.03
Current smoker (some days)	2.89 (1.35-6.17)	
Former smoker	1.16 (0.80-1.67)	
Never smoked	1.00 (ref)	

a In this table, all characteristics presented in Table 2 were further evaluated in a multivariable logistic regression model to determine which characteristics retained significant associations with whether or not a pregnant woman received dental care in the last 12 months. Only annual income and smoking were significant at P ≤ .05. CI = confidence interval; ref = reference group.
